# Regarding the role of metabolite on the risk for severe postoperative complications in colorectal cancer patient

**DOI:** 10.1097/JS9.0000000000001087

**Published:** 2024-01-18

**Authors:** Wei Yang, Jinlin Liu

**Affiliations:** a Department of Clinical Laboratory, Zhejiang Provincial People’s Hospital Bijie Hospital, Bijie, Guizhou; b Department of Clinical Laboratory, South China Hospital, Medical School, Shenzhen University, Shenzhen, People’s Republic of China

*Dear Editor*,

We read Blanca’s study^1^ with great interest. In this study, the author collected plasma samples before and after colorectal cancer surgery in 146 patients, and analyzed 188 metabolites and 21 ratios by mass spectrometry. They found the strongest positive relationship with both Clavien–Dindo and Comprehensive Complication Index with kynurenine/tryptophan, an inverse relationship with lysophosphatidylcholines and phosphatidylcholines. Hexadecanoylcarnitine below 0.093 µM had a 12-fold higher risk of anastomotic leakage-related complications. Then, they concluded that surgery-induced phospholipids and amino acid dysregulation are associated with the severity of postoperative complications after colorectal cancer surgery. Despite definite results, we raise some concerns about the result of hexadecanoylcarnitine and other comments about this study.

Hexadecanoylcarnitine or palmitoylcarnitine is an acylcarnitine^2^. It is a long-chain acyl fatty acid derivative ester of carnitine, which facilitates the transfer of long-chain fatty acids from cytoplasm into mitochondria during the oxidation of fatty acids. The general role of acylcarnitines is to produce energy^2^. Of the long-chain carnitine esters, significantly higher hexadecanoylcarnitine was noted in the newly diagnosed type 2 diabetes (T2D) group or pre-diabetes group when compared to the normal glucose tolerance subjects^3^. Recently, more and more studies indicated that hexadecanoylcarnitine was associated with higher T2D risk^4^. However, in Blanca’s study, there are 2 type 1 diabetes patients and 39 T2D patients among 146 colorectal cancer surgery patients. Thus, it is challenging to pinpoint the specific association without the consideration of the diabetes confounder. The patient’s diabetes comorbidities may have contributed to dysregulated hexadecanoylcarnitine. Therefore, without statistical analysis this diabetes confounder, will result in inappropriate statical results, although the author used the before and after surgery for the comparison.

Second, another important factor may also be ignored, which also greatly influences the results of plasma metabolites results, that is the commonly used chemotherapy drugs for colorectal cancer patients including 5-fluorouracil, capecitabine, irinotecan, oxaliplatin or trifluridine and tipiracil. They all had a great influence on the plasma metabolites in these colorectal cancer patients. However, the drug use history or their statistical significance in Khalid’s study were not listed, which could result in inappropriate results.

Thus, in this letter, we want to discuss whether a clear association between anastomotic leakage-related complications or other postoperative complications in colorectal cancer cases with these metabolic markers exists. However, although we felt the need to address these few points, we appreciate Blanca’s paper^1^ giving this opportunity to discuss the potential early metabolomics signatures capable of detecting patients at risk for severe postoperative complications after colorectal cancer surgery.

## Ethical approval

Not applicable.

## Consent

Not applicable.

## Sources of funding

Not applicable.

## Author contribution

W.Y. and J.L.: discussed and wrote the manuscript.

## Conflicts of interest disclosure

We declare no conflicts of interest.

## Research registration unique identifying number (UIN)

Not applicable.

## Guarantor

Jinlin Liu.

## Data availability statement

Not applicable.

## Provenance and peer review

Not applicable.
